# Reactions in the Photocatalytic Conversion of Tertiary Alcohols on Rutile TiO_2_(110)

**DOI:** 10.1002/anie.201907917

**Published:** 2019-08-28

**Authors:** Carla Courtois, Moritz Eder, Kordula Schnabl, Constantin A. Walenta, Martin Tschurl, Ulrich Heiz

**Affiliations:** ^1^ Chair of Physical Chemistry Department of Chemistry & Catalysis Research Center Technische Universität München Lichtenbergstraße 4 85748 Garching Germany

**Keywords:** alcohol reforming, photocatalysis, reaction mechanisms, tertiary alcohols, titania

## Abstract

According to textbooks, tertiary alcohols are inert towards oxidation. The photocatalysis of tertiary alcohols under highly defined vacuum conditions on a titania single crystal reveals unexpected and new reactions, which can be described as disproportionation into an alkane and the respective ketone. In contrast to primary and secondary alcohols, in tertiary alcohols the absence of an α‐H leads to a C−C‐bond cleavage instead of the common abstraction of hydrogen. Surprisingly, bonds to methyl groups are not cleaved when the alcohol exhibits longer alkyl chains in the α‐position to the hydroxyl group. The presence of platinum loadings not only increases the reaction rate but also opens up a new reaction channel: the formation of molecular hydrogen and a long‐chain alkane resulting from recombination of two alkyl moieties. This work demonstrates that new synthetic routes may become possible by introducing photocatalytic reaction steps in which the co‐catalysts may also play a decisive role.

## Graphical abstract

**Breaking ground**: The photoreforming of tertiary alcohols on TiO_2_(110) follows an unexpected reaction channel, selective disproportionation to an alkane and the respective ketone. When the catalyst is decorated with Pt clusters, the overall reaction rate increases. In addition to ketone formation, a new reaction pathway is observed, hydrogen evolution and alkane dimerization.

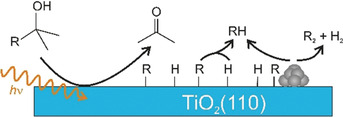

The selective oxidation of alcohols to aldehydes and ketones is a fundamental topic in various fields of chemistry ranging from heterogeneous catalysis to synthetic organic chemistry.^[1]^ In contrast to the facile oxidation of primary and secondary alcohols, tertiary alcoholstypically do not react analogously, due to the required cleavage of a C−C instead of a C−H bond to establish the carbonyl functionality.^[2]^ As tertiary alcohol oxidation is generally difficult, in particular in a selective manner, publications on this subject are thus scarce and often a broad product spectrum results.[^1c^, ^2^, ^3^] Conventional synthetic methods often rely on auxiliary compounds or quantitative amounts of oxidants to enable the reaction in the first place.^[4]^ Often the conversion is conducted with the use of toxic metal oxides such as chromium(VI) oxides.^[5]^

An alternative approach for alcohol reforming is photocatalysis using semiconductors.^[6]^ For example, Teichner and co‐workers successfully photooxidized 2‐methyl‐2‐butanol by means of UV irradiation on a nonporous anatase catalyst in the presence of oxygen. The proposed reaction pathways take place via olefin intermediates, leading to the reaction products acetone, ethanal, and 2‐butanone.^[7]^

In general, titania is by far the most used material in photocatalysis due to its reaction properties and availability.^[8]^ While it is commonly applied in a nanostructure form (e.g. as P25), the material's structural complexity often prevents the elucidation of exact reaction mechanisms.^[9]^ As in thermal catalysis, defined single‐crystal surfaces under highly defined conditions in ultra‐high vacuum (UHV) are more suitable for this purpose.^[10]^ In heterogeneous photocatalysis, rutile TiO_2_(110) surfaces have been comprehensively employed in alcohol conversion thermally^[11]^ and photochemically.^[12]^ Thus, this material represents the best‐suited model system, even though other titania systems (e.g. anatase) may exhibit better photoactivities.

For this report, we investigated the photochemical reaction behavior of longer‐chain tertiary alcohols (3‐methyl‐3‐hexanol, 2‐methyl‐2‐pentanol, and 2‐methyl‐2‐butanol) on bare and platinum‐loaded rutile TiO_2_(110) in an UHV environment in the absence of oxygen and water. We demonstrate that the alcohols undergo unexpected and new photocatalytic reactions, which enable general mechanistic insights. Furthermore, we show that the rich chemistry of tertiary alcohols makes them an interesting model system for photocatalysis. For example, they enable the elucidation of the behavior of alkyl radicals on surfaces, important for the photo‐Kolbe reaction^[13]^ and the Fischer–Tropsch process.^[14]^ In the latter, TiO_2_ represents a common support material.^[15]^

The UV illumination of a TiO_2_(110) crystal, decorated with a defined coverage (0.1 % monolayer (ML)) of platinum clusters ranging in size range from Pt_8_ to about Pt_25_, leads to a photocatalytic reaction of 3‐methyl‐3‐hexanol, which results in a complex fragmentation pattern in the mass spectrum. However, a detailed analysis reveals (see Figure S2) that only two parallel reactions occur, both of which are an oxidation to a ketone and a corresponding alkane (2‐pentanone and ethane, 2‐butanone and propane, see Scheme 1). While the formation of higher alkanes is observed, a reaction yielding methane is not. Monitoring mass traces specific for a particular molecule (Figure 1) demonstrates that the reaction is truly catalytic under illumination and formation of unwanted surface species leading to catalyst poisoning does not occur. Compared to the photoreforming of other alcohols, the observed reaction pathways are unexpected. The absence of an *α*‐H precludes the common C−H cleavage to form H_2_ and the respective aldehyde or ketone, as detected for primary and secondary alcohols.[^2^, ^16^] Therefore, the ejection of radicals and concomitant stoichiometric production of H_2_ is expected in analogy to *tert*‐butanol photoreforming.^[17]^ Neither radical abstraction nor significant molecular hydrogen formation is detected for prolonged reaction times. Instead, this reaction, which to the best of our knowledge has never been described before, can be viewed as a photocatalytic disproportionation yielding higher alkanes and the respective ketones. Interestingly, reaction products originating from the cleavage of the methyl group are not observed (Figure S9). In the same way, dehydration reactions, common in thermal reactions, also do not occur (Figure S9). The same reaction is observed for tertiary alcohols with two methyl groups at the *α‐*C position (namely, 2‐methyl‐2‐butanol and 2‐methyl‐2‐pentanol), for which only the long carbon chain is abstracted with 100 % selectivity (see Figures S3 and S4). Consequently, the formation of acetone and the respective alkane results exclusively. This demonstrates the generality of our findings. The same products are observed under ambient conditions even for 2‐methy‐2‐butanol, but the presence of oxygen and water leads to additional by‐products.[^7^, ^18^]

**Figure 1:**
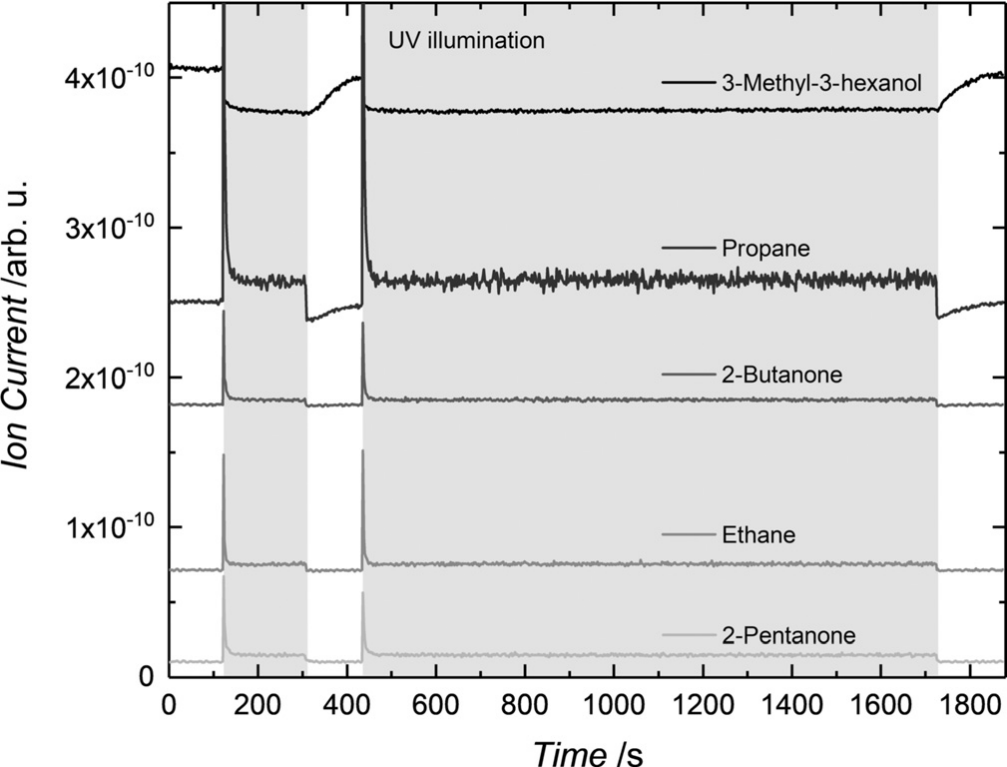
Photocatalytic products of 3‐methyl‐3‐hexanol photoreforming on Pt_*x*_/r‐TiO_2_(110) (0.1 % monolayer (ML) cluster coverage). Signals for 3‐methyl‐3‐hexanol (*m*/*z* 73), propane (*m*/*z* 29), 2‐butanone (*m*/*z* 72), ethane (*m*/*z* 30), and 2‐pentanone (*m*/*z* 86) are shown at 340 K under a 3‐methyl‐3‐hexanol background pressure of 1.7×10^−7^ mbar. The gray region highlights the period of UV laser irradiation. The initial burst of the signal originates from higher surface concentrations of the alcohol before the start of the illumination. Note that the traces are offset for clarity. The traces demonstrate that two different photocatalytic reactions occur in parallel yielding a ketone and the respective alkane.

**Scheme 1:**
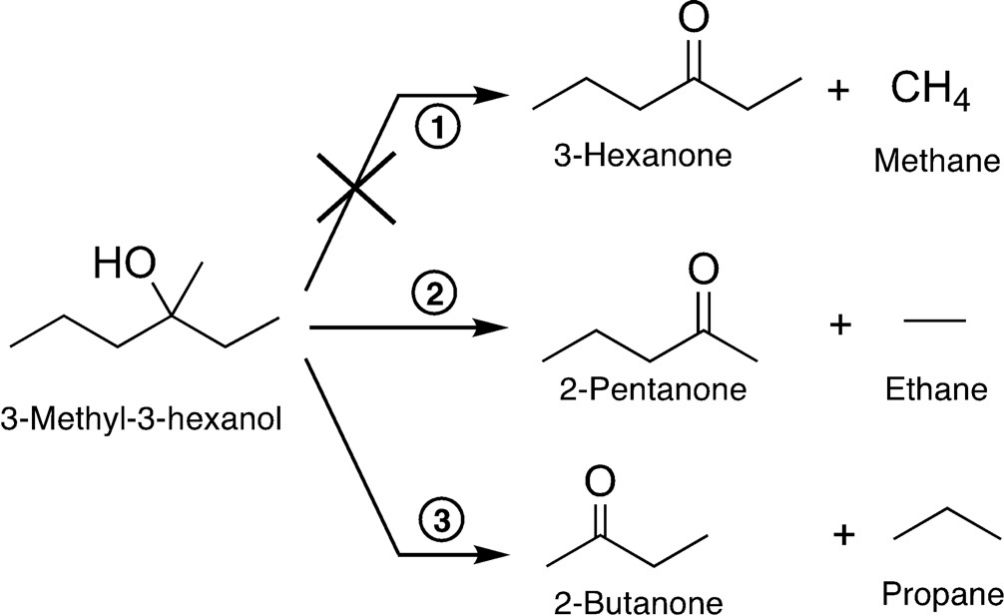
Reaction scheme for the photoreforming of 3‐methyl‐3‐hexanol on Pt_*x*_/r‐TiO_2_(110) and on r‐TiO_2_(110) under UV illumination. The reaction can be seen formally as a hole‐mediated disproportionation yielding an alkane and the respective ketone; however, this does not occur for the formation of methane.

This new reaction can be explained with the mechanism we suggested for the photoreforming of alcohols on TiO_2_ in the gas phase.^[17a]^ The photoactive alkoxy species, which are already formed upon surface adsorption in the dark[^12^, ^19^] undergo a hole‐mediated oxidation reaction, resulting in the cleavage of a C−C bond. The role of alkoxy compounds as the photoactive species on TiO_2_(110) in alcohol photoreforming has been demonstrated convincingly in the works of Henderson and others.[^12^, ^20^] Methyl radical ejection observed with *tert*‐butanol demonstrates that the photocatalytic oxidation reaction occurs via a homolytic C−C bond scission. In contrast to methyl groups, longer alkyl chains such as ethyl and propyl exhibit stronger interactions with the TiO_2_ surface in their adsorption geometry and thus remain on the surface. This is in perfect agreement with their absence in the mass spectra.

These surface alkyl radicals undergo recombination in a consecutive thermal reaction step with hydrogen atoms originating from the dissociative adsorption of the alcohol. This reaction is also facilitated on bare TiO_2_(110) (i.e., in the absence of Pt) in contrast to the recombination of two hydrogen atoms. Consequently, photoreforming of higher tertiary alcohols occurs in a photocatalytic manner even without any co‐catalyst (Figure 2 a) and on a hydroxylated surface (as shown in Figure S13 for 2‐methyl‐2‐pentanol photoreforming), in contrast to α‐H‐containing alcohols. For the latter, surface hydroxylation results in the poisoning of the photocatalyst.^[17a]^ The deposition of small amounts of Pt clusters significantly increases the overall reaction rate, with higher loadings leading only to a small increase in the turnover frequency (TOF) (Figure 2 a). This trend is in good agreement with findings from methanol photoreforming in UHV^[17a]^ and with colloidal systems.^[21]^

**Figure 2:**
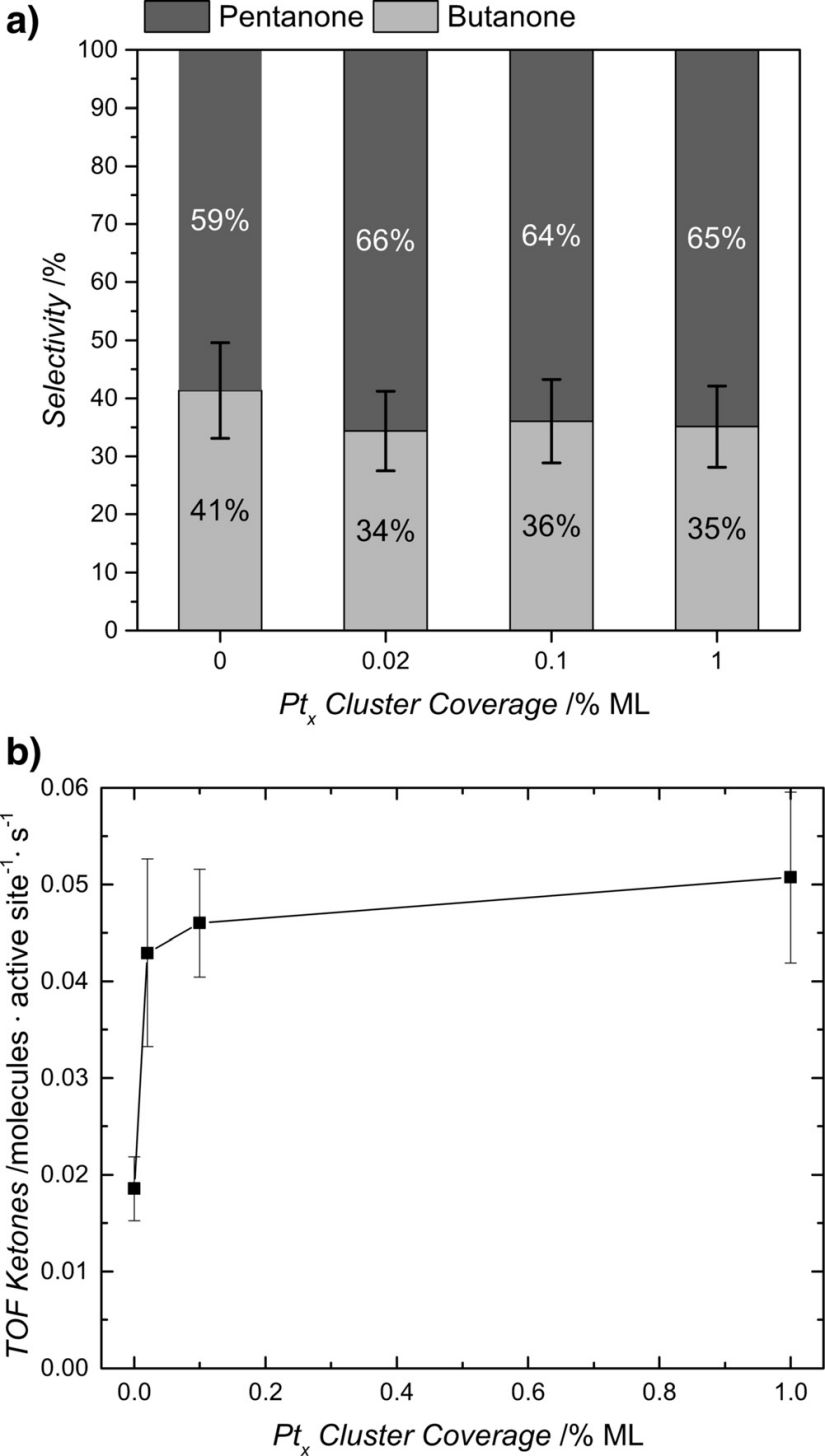
Photocatalytic conversion of 3‐methyl‐3‐hexanol on Pt‐decorated r‐TiO_2_(110). In (a) the TOF of the ketones (sum of 2‐butanone and 2‐pentanone) is shown for different Pt_*x*_ cluster coverages. In (b) the selectivities for 2‐pentanone and 2‐butanone based on the TOFs are displayed for bare r‐TiO_2_(110) and for different Pt loadings on r‐TiO_2_(110). A monolayer ML refers to the surface atoms. 0 % ML stands for the bare r‐TiO_2_(110). While the deposition of Pt clusters does not affect the reaction's selectivity, it initially increases the TOF. However, higher loadings do not have a similar effect.

In good accordance with the interpretation of the photooxidation on the semiconductor and a consecutive alkane formation, the selectivity of the reaction of 3‐methyl‐3‐hexanol remains unaffected by the degree of Pt coverage (Figure 2 b). It also remains constant at temperatures (Figure S8 b) between 230 and 360 K, further demonstrating the photocatalytic nature of the reaction. In order to explain the observed selectivity of pentanone to butanone of about 2:1 (i.e., the preferred cleavage of ethyl over propyl and the general absence of methyl), the thermochemistry of the reactions may be used to obtain qualitative insights. All three possible reactions displayed in Scheme 1 are endothermic by about 20 to 30 kJ mol^−1^ (see the corresponding chapter in the Supporting Information). Model reactions for the photocatalytic C−C bond cleavage suggest that the formation of methyl radicals requires significantly more energy than ethyl or propyl formation (see details in the Supporting Information). In addition, longer‐chain alkyl moieties than methyl exhibit stronger interactions with the surface. These radicals are therefore not detected in the gas phase, in contrast to the ejection of methyl radicals in *tert*‐butanol photoreforming. For ethyl and propyl formation, the difference in thermochemistry is less pronounced compared to methyl. However, reactions yielding ethyl are generally more endothermic than propyl formation. This trend is reflected in the observed selectivity of the reaction; pentanone and an ethyl radical are preferentially formed over butanone and a propyl radical. Therefore, thermodynamic values may be used as a rule‐of‐thumb to predict preferential bond cleavage in similar photoreactions.

Performing the reaction at different pressures (3×10^−8^ mbar to 5×10^−6^ mbar) with the Pt‐loaded photocatalyst does not affect the branching ratio for the two reactions (Figure S8 a). The overall TOFs exhibit typical 1^st^ order behavior when the reaction is limited by reactant adsorption and 0^th^ order in the case of limitation by product desorption (see Figures S5 and S6). Similarly, the illumination‐dependent TOFs (see Figure S7) suggest a first‐order behavior at lower irradiation illumination intensities, which transfer into a saturation regime (zeroth order) at higher photon fluxes, as in the photoreforming of other alcohols.^[17a]^ However, and more importantly, for platinum‐decorated TiO_2_(110) an additional side reaction becomes evident at higher pressures. This is best illustrated for 2‐methyl‐2‐pentanol photoreforming (Scheme 2), for which all reaction products can clearly be quantified and their analysis is not affected by isobaric interference. As deposited platinum clusters enable the efficient thermal recombination of hydrogen atoms,^[17a]^ the surface coverage of alkyl increases in the steady state with increasing pressure. Consequently, the recombination product of two radicals (i.e., hexane) accompanied by H_2_ formation is detected at 5×10^−6^ mbar of alcohol pressure (Figure 3 a).

**Figure 3:**
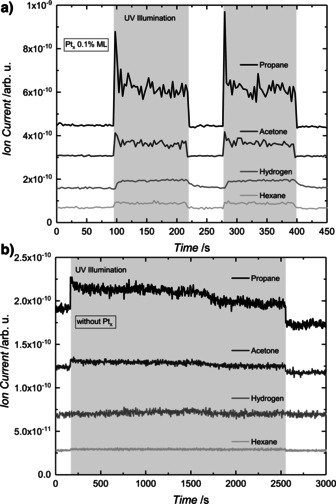
Photocatalytic products of 2‐methyl‐2‐pentanol photoreforming on a) Pt_*x*_/r‐TiO_2_(110) (0.1 % ML cluster coverage) and b) r‐TiO_2_(110). Propane (*m*/*z* 29), acetone (*m*/*z* 58), hydrogen (*m*/*z* 2), and hexane (*m*/*z* 86) signals are shown at 336 K under a 2‐methyl‐2‐pentanol background pressure of 5.0×10^−6^ mbar. The gray region highlights the period of UV laser irradiation. Note that the traces are offset for clarity. In contrast to bare TiO_2_(110), the co‐catalyst‐loaded semiconductor enables another side reaction, alkyl radical recombination and molecular hydrogen formation.

**Scheme 2:**
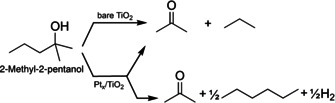
Reaction scheme for the photoreforming of 2‐methyl‐2‐pentanol on bare r‐TiO_2_(110) and Pt_*x*_/r‐TiO_2_(110) under UV illumination. While on bare titania only the hole‐mediated disproportionation yielding acetone and propane occurs, a second reaction pathway is enabled for Pt‐decorated TiO_2_ above 2.0×10^−7^ mbar alcohol pressure. In the latter reaction, hydrogen recombines on the Pt clusters and two propyl radicals recombine forming hexane.

As the formation of H_2_ is not facilitated on bare titania, this side reaction is not observed in the absence of a co‐catalyst (Figure 3 b). Consequently, this result also demonstrates that with the addition of noble‐metal clusters, not only unwanted consecutive reactions (as for example the hydrogenation of ketones recently studied mechanistically by electrochemistry^[22]^), but also an intrinsically different outcome of the photoreaction cycle must be considered in applied systems.

To summarize, we discovered a new reaction for the photoreforming of tertiary alcohols on rutile, which can be described as hole‐mediated disproportionation yielding an alkane and the respective ketone. Surprisingly, the abstraction of methyl groups does not occur and only α‐C bonds to longer alkyl chains are selectively cleaved, in contrast to the reaction of *tert*‐butanol. The thermochemistry of radical formation may supply a qualitative measure to predict the selectivity of the photoreaction. As the recombination of the alkyl radical and hydrogen is enabled on bare titania, in contrast to the recombination of two hydrogen atoms, the reaction is fully catalytic even without a co‐catalyst. While already small amounts of Pt clusters on the rutile crystal increase the overall reaction rate, they also induce another reaction pathway yielding molecular hydrogen and the recombination product of two radicals observed at increasing pressures.

The observed mechanisms may explain the variety of product distributions from ambient pressure and liquid photoreforming studies and have set mechanistic research in photocatalysis on a solid foundation.

## Conflict of interest

The authors declare no conflict of interest.

## Supporting information

As a service to our authors and readers, this journal provides supporting information supplied by the authors. Such materials are peer reviewed and may be re‐organized for online delivery, but are not copy‐edited or typeset. Technical support issues arising from supporting information (other than missing files) should be addressed to the authors.

Supplementary
