# Supplementary material for: Reactions in the Photocatalytic Conversion of Tertiary Alcohols on Rutile TiO2(110)
Source: Angew Chem Int Ed Engl. 2019 Aug 28;58(40):14255–9. doi: 10.1002/anie.201907917 (PMC7687105; doi:10.1002/anie.201907917)
Supplement: Supplementary file 1 — Supplementary [file ANIE-58-14255-s001.pdf]

Angewandte Chemie
Eine Zeitschrift der Gesellschaft Deutscher Chemiker

Supporting Information

Reactions in the Photocatalytic Conversion of Tertiary Alcohols on Rutile TiO_2(110)

*Carla Courtois[+], Moritz Eder[+], Kordula Schnabl, Constantin A. Walenta, Martin Tschurl, and Ulrich Heiz**

anie_201907917_sm_miscellaneous_information.pdf

Supporting Information:

Experimental Overview

The details of the experimental setup are described below and also elsewhere.[S1] In brief, measurements are performed with a complex UHV apparatus featuring a laser vaporization cluster source for the deposition of clusters with an atomically precise number of atoms.[S2] The cluster coverage is controlled by the deposition time and determined by recording the neutralization current with a picoammeter. The UHV chamber is equipped with an Auger spectrometer and an ion gun for Ar^+ sputtering for the preparation of a defined semiconductor surface. The sample is mounted on a sample holder,[S3] which is attached to a heatable and

liquid-N_2-coolable manipulator in order to set the sample on a selected and defined temperature. The $TiO_2(110)$ crystal is cleaned following established procedures of sputtering and annealing cycles. The degree of surface reduction and absence of platinum is determined by H_2O temperature-programmed desorption and the evaluation of traces of water[S4] and H_2,[S5] respectively. The Pt/$TiO_2(110)$ model catalysts have been thoroughly characterized by a variety of local and integral techniques.[S6] Photocatalytic measurements are performed by illuminating the sample with a Nd:YAG-pumped, frequency-doubled OPO laser beam at 242 nm in an alcohol background. Product evolution is followed by a quadrupole mass spectrometer placed in line of sight with the photocatalyst.

Experimental Details

The setup consists of a laser vaporization cluster generation source and an ultra-high vacuum (UHV) setup. For cluster generation, a focused beam of the frequency-doubled of a Nd:YAG (532 nm, 100 Hz, Spitlight DPSS, Innolas) ablates a rotating Pt target (99.96% purity, ESG Edelmetalle, Germany). The resulting plasma is cooled by the expansion of a He gas pulse (He 6.0, Air Westfalen) into the vacuum. The cationic cluster beam is guided and bent into a quadrupole mass filter (QMF; Extrel, USA), which enables either the selection of a particular cluster size or the guidance of the clusters in ion-guide mode.[S2] For this study, the latter mode was used and was operated as high-pass filter transmitting only ions larger than Pt_7. The settings resulted in a cluster size distribution with a maximum from Pt_{11} to Pt_{13} (see S1). 0.1% monolayer (ML) of Pt_x clusters (respective to surface atoms) were deposited onto a $TiO_2(110)$ single crystal under soft landing conditions (<1eV/atom in kinetic energy). Cluster loadings were determined by recording the cluster neutralization current during the deposition with a picoammeter (Keithley, 6587). For experiments with different cluster coverages, the desired amount of platinum, which is specified in the presented data, was deposited by varying the deposition time (in the order of minutes). The as-obtained Pt-

decorated $TiO_2(110)$ catalysts have been been well-characterized in previous works by means of scanning probe microscopy and photoelectron spectroscopy.[S6–S9]

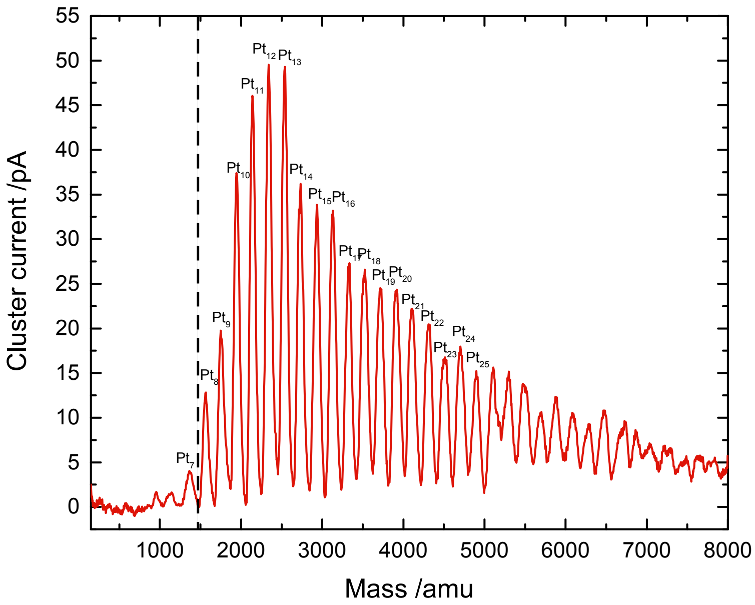

Figure S1: Size-distribution of platinum clusters from Pt_7 to Pt_{35} with a maximum from Pt_{11} to Pt_{13} from the laser vaporization cluster source. The solid black line denotes the cut-off mass of the quadrupole mass filter during deposition.

In the UHV setup, a base pressure lower than $9.8 \cdot 10^{-11}$ mbar is achieved. The sample in the chamber is mounted on a sample holder,[S3] which is attached to a (ϕ,x,y,z)-manipulator (VAB Vakuum GmbH) in order to enable the movement of the sample to different positions. The sample holder enables liquid nitrogen cooling and the resistive heating of the crystal. For analysis, an auger spectroscope (MDC, HLM-275-3), an electron ionization quadrupole mass spectrometer (EI-QMS; QMA 430, Pfeiffer Vacuum GmbH) and a home-built photoionization time-of-flight mass spectrometer (PI-TOF-MS) are attached to the main chamber. The chamber is further equipped with a leak valve (Pfeiffer Vacuum) for Langmuir dosing and a molecular beam doser in order introduce reactant gases into the vacuum via a gas line. The vapor pressure of 3-methyl-3-hexanol (99%, Alfa Aesar), 2-methyl-2-pentanol (99%, Sigma Aldrich) and 2-methyl-2-butanol ($\geq$ 99%, Sigma Aldrich) is exploited in order to introduce the reactants in the reaction chamber via a leak valve twith a constant background pressure.

The surface of the rutile $TiO_2(110)$ single crystal (SurfaceNet GmbH) is prepared by cycles of Ar^+ (100% N60; Air Liquide) sputtering (1 keV, $4 \cdot 10^{-5}$ mbar for several hours), oxygen ($\geq$ 99%; Westfalen) annealing (800 K, $1 \cdot 10^{-6}$ mbar, 20 min) and vacuum annealing (800 K, 10 min) until no contamination is observed by Auger electron spectroscopy with a respective spectrometer (Omicron Nanotechnology). The absence of Pt is further confirmed by the evaluation of the H_2 trace in a H_2O thermal programmed desorption (TPD) experiment.[S5] The resulting light blue TiO_2 has a bridge-bonded oxygen (BBO) vacancy concentration of $6 \pm 1\%$ of Ti lattice sites, which is determined by H_2O TPD.[S4] All the experiments are performed on such a reduced $TiO_2(110)$ crystal. Photoexcitation experiments are conducted with a frequency doubled OPO laser (GWU, premiScan ULD/400), which is pumped with the third harmonic of a Nd:YAG (Innolas Spitlight HighPower 1200, 7 ns pulse width, 20 Hz repetition rate), in order to achieve a wavelength of 242 nm (with a power of 3.6 ± 0.3 mW at the crystal surface, if not otherwise noted). Product identification is performed with the above-mentioned QMS with mass scans under catalytic conditions (see for example figure S2) and the recording of specific mass traces for the quantification of the reaction rates (see for example figure S11). The QMS ion current is calibrated via the desorption integral of a saturation coverage of the Ti-lattice sites with methanol in a TPD experiment.

The turnover frequency (TOF) values are calculated by integrating the baseline corrected signals of the QMS. These are further corrected with the m/z-dependent transmissions through the QMS, electron impact ionization cross sections (ICS) as well as with a factor considering ion fragmentation which are taken from reference mass spectra. The fragmentation pattern are obtained from recording the mass spectra of the respective molecules. The ICS values and the m/z-value of the respective fragment used for the quantification are given in table S1.

Table S1: Electron impact ionization cross sensitivity values at 70 eV and m/z-value of the respective fragment used for quantification.

substance	ICS [Å^2]	m/z fragment
2-butanone	12.9[S10]	72
2-pentanone	15.2[S10]	86
propane	11.6[S10]	29
ethane	8.39[S10]	30
acetone	10.2[S10]	58
hydrogen	1.021[S11]	2
hexane	20.8[S10]	86

Evaluation of Mass Spectra

The products are identified by the evaluation the mass scans. Firstly, a mass scan **I** over the whole mass range with potential product masses, is carried out at a certain alcohol background pressure, which is given in the caption of the respective figure. This mass scan **I** includes the fragmentation pattern of the alcohol and possible contaminations from the residual gas in the UHV chamber (mainly H_2, CO, CO_2 and H_2O). Secondly, the sample is illuminated and a mass scan **II** under steady-state condition is recorded. This mass scan **II** includes the fragmentation pattern of the alcohol and its photo products. Subtracting mass scan **I** (dark) from mass scan **II** (illuminated) reveals a mass scan of the photocatalytic reaction. These spectra are named as difference spectra an are shown in Figure S2a, S3a and S4a. Positive values indicate that these masses arise from photo products and negative values originate from the consumed alcohol. In order to demonstrate that the resulting mass peaks are due to the presence of the ketones and the respective alkanes, self-recorded mass spectra of the reactant and products are added or subtracted from the difference spectrum

a).

In a first step, from spectrum **a)** to **b)**, the mass spectrum of the alcohol is added to the difference spectrum. This results spectrum **b)**, which only includes reaction products (i.e. positive signals). Subsequently, the mass peaks of one product after the other is subtracted from **b)** so that a baseline spectrum **d)** is obtained. Before every addition or subtraction, the mass spectrum is normalized to a unique mass fragment of the respective molecule. All the products can be clearly identified and it can be demonstrated, that no other products result.

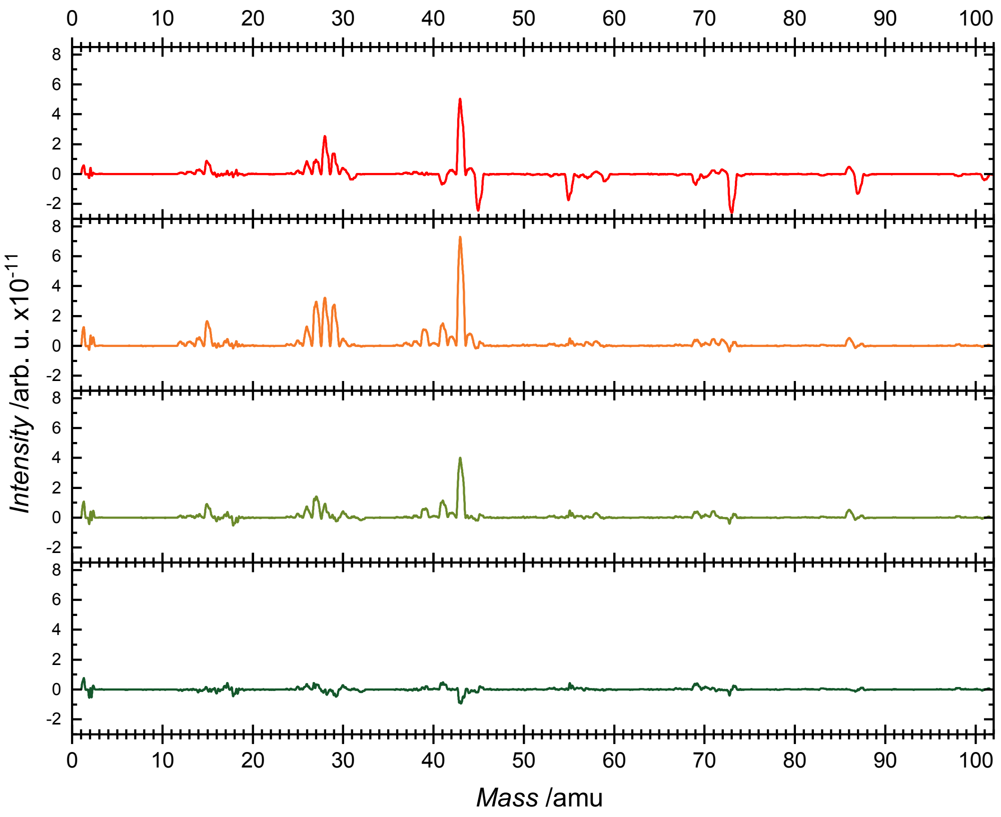

Figure S2: Mass spectra for the product analysis of the photocatalytic reforming of 3-methyl-3-hexanol (0.1% ML Pt_x/TiO_2, 300 K, alcohol background pressure $1.7 \cdot 10^{-7}$ mbar). The difference spectrum is shown in **a)**. Positive peaks originate from the products (2-butanone, propane, 2-pentanone and ethane), and the negative peaks stem from consumed 3-methyl-3-hexanol. Spectrum **b)** is obtained by adding the mass spectrum of 3-methyl-3-hexanol to the difference spectrum (both spectra were normalized to $m/z = 73$ prior to the addition). **c)** depicts the mass spectrum after the subtraction of the spectra of 2-butanone (normalized to $m/z = 72$) and propane (normalized to $m/z = 44$). Spectrum **d)** shows a baseline after subtracting the spectra of 2-pentanone (normalized to $m/z = 86$) and ethane (normalized to $m/z = 28$) from spectrum **c)**.

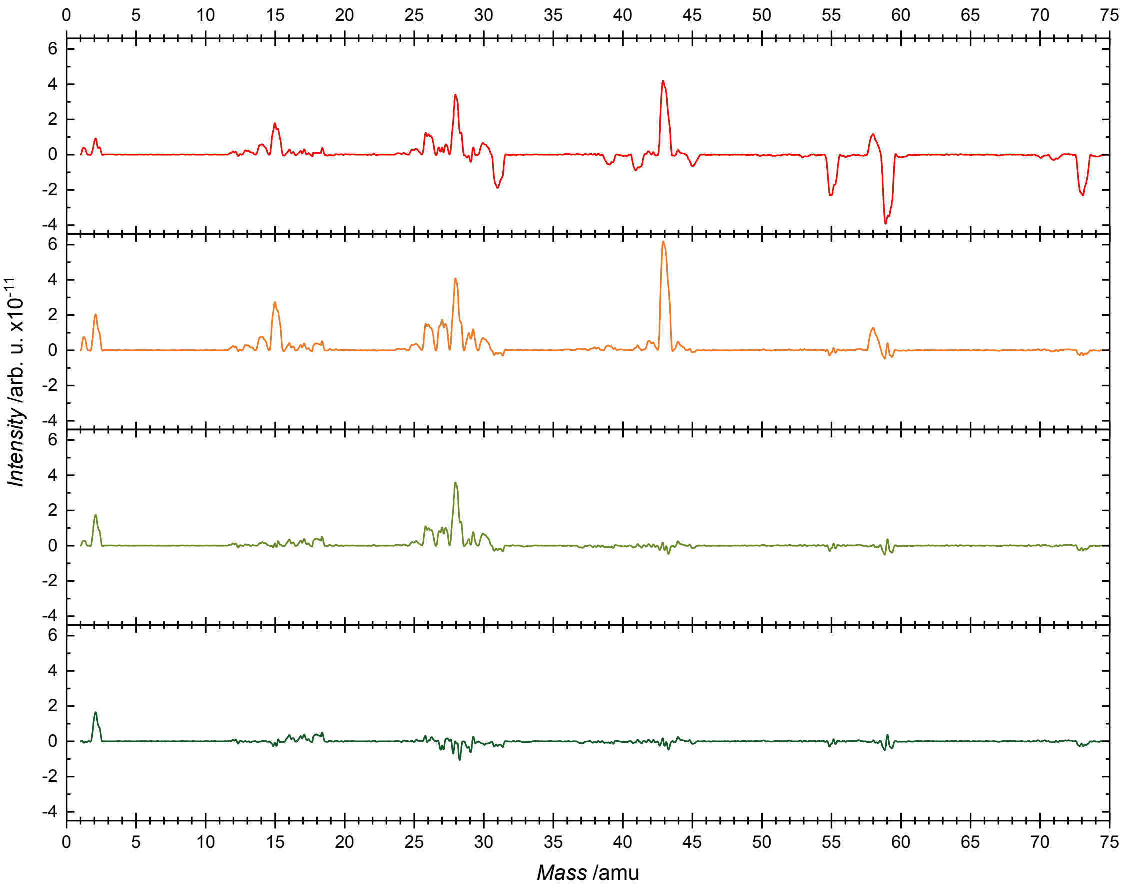

Figure S3: Mass spectra for the product analysis of the photocatalytic reforming of 2-methyl-2-butanol (0.1% ML Pt_x/TiO_2, 263 K, alcohol background pressure $2.0 \cdot 10^{-7}$ mbar). The difference spectrum is shown in **a)**. Positive peaks originate from the products (acetone and ethane), and the negative peaks stem from consumed 2-methyl-2-butanol. Spectrum **b)** is obtained by adding the mass spectrum of 2-methyl-2-butanol to the difference spectrum (both spectra were normalized to $m/z = 59$ prior to the addition). **c)** depicts the mass spectrum after the subtraction of the spectrum of acetone (normalized to $m/z = 43$). Spectrum **d)** shows a baseline after subtracting the spectrum of ethane (normalized to $m/z = 28$) from spectrum **c)**.

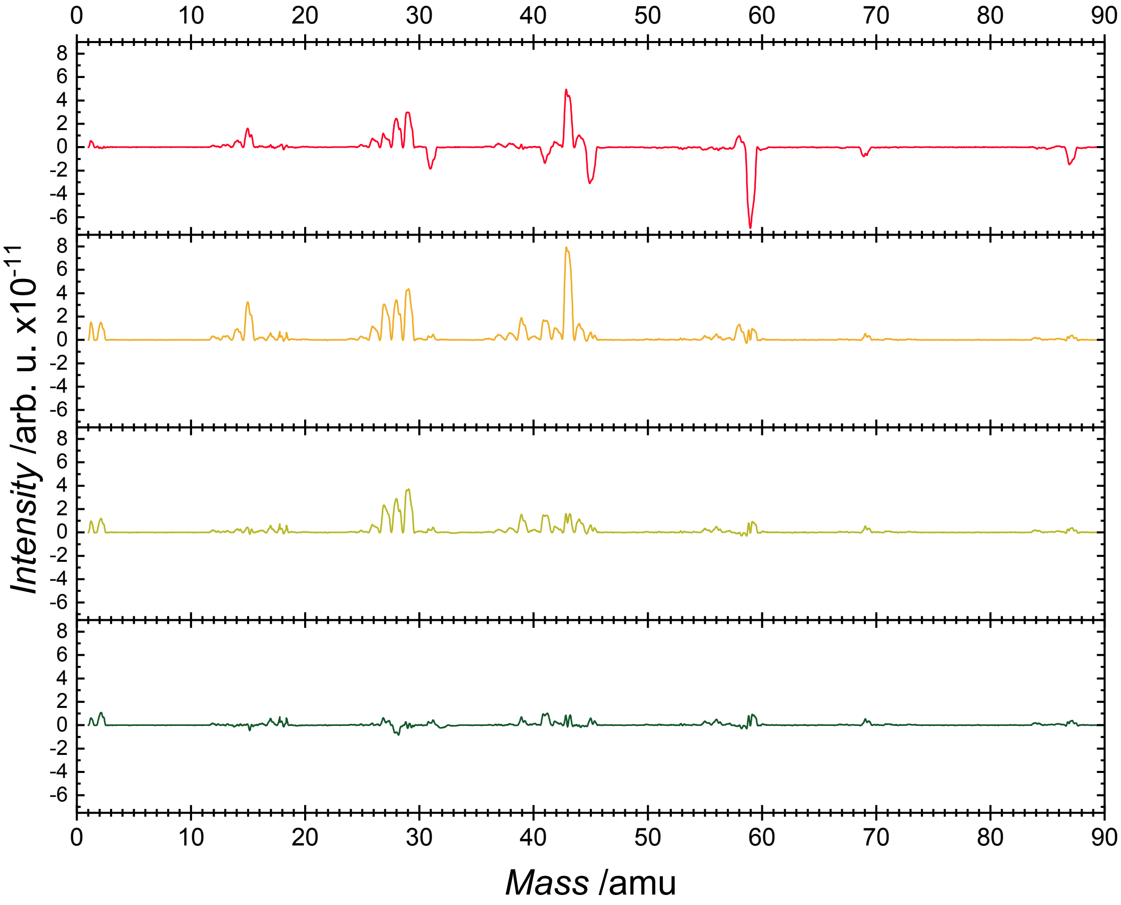

Figure S4: Mass spectra for the product analysis of the photocatalytic reforming of 2-methyl-2-pentanol (0.1% ML Pt_x/TiO_2, 321 K, alcohol background pressure $2.0 \cdot 10^{-7}$ mbar). The difference spectrum is shown in **a)**. Positive peaks originate from the products (acetone and propane), and the negative peaks stem from consumed 2-methyl-2-pentanol. Spectrum **b)** is obtained by adding the mass spectrum of 2-methyl-2-pentanol to the difference spectrum (both spectra were normalized to $m/z = 59$ prior to the addition). **c)** depicts the mass spectrum after the subtraction of the spectrum of acetone (normalized to $m/z = 58$). Spectrum **d)** shows a baseline after subtracting the spectrum of ethane (normalized to $m/z = 29$) from spectrum **c)**.

Turnover Frequencies

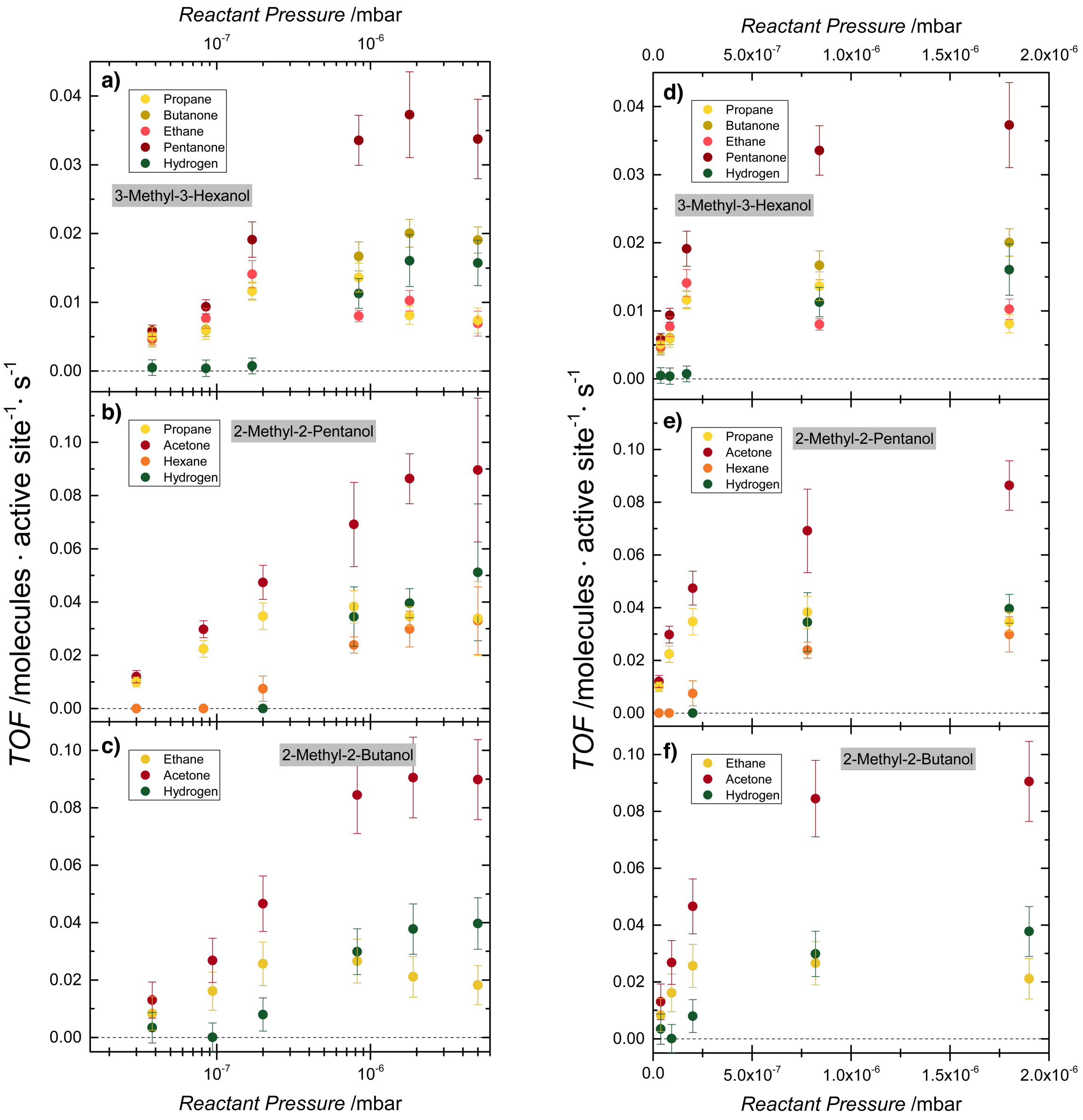

Figure S5: Pressure-dependent Turnover Frequencies (TOFs) for photocatalytic reforming of **a)** and **d)** 3-methyl-3-hexanol, **b)** and **e)** 2-methyl-2-pentanol, **c)** and **f)** 2-methyl-2-butanol over 0.1% ML Pt_x/TiO_2. The photocatalytic experiments are performed at 253 K, so that the temperature is above the desorption temperature of the ketones and alkanes and below the desorption temperature of the alcohol. The TOF exhibit a 1st order behavior until the regime changes from reactant adsorption to product desorption limitation. The latter results in a 0st order behavior. In **a)**, **b)** and **c)**, the pressure-dependent TOFs are plotted on a logarithmic scale and in **d)**, **e)** and **f)**, a section of the TOFs are plotted linearly to demonstrate a linear rise before the saturation behaviour.

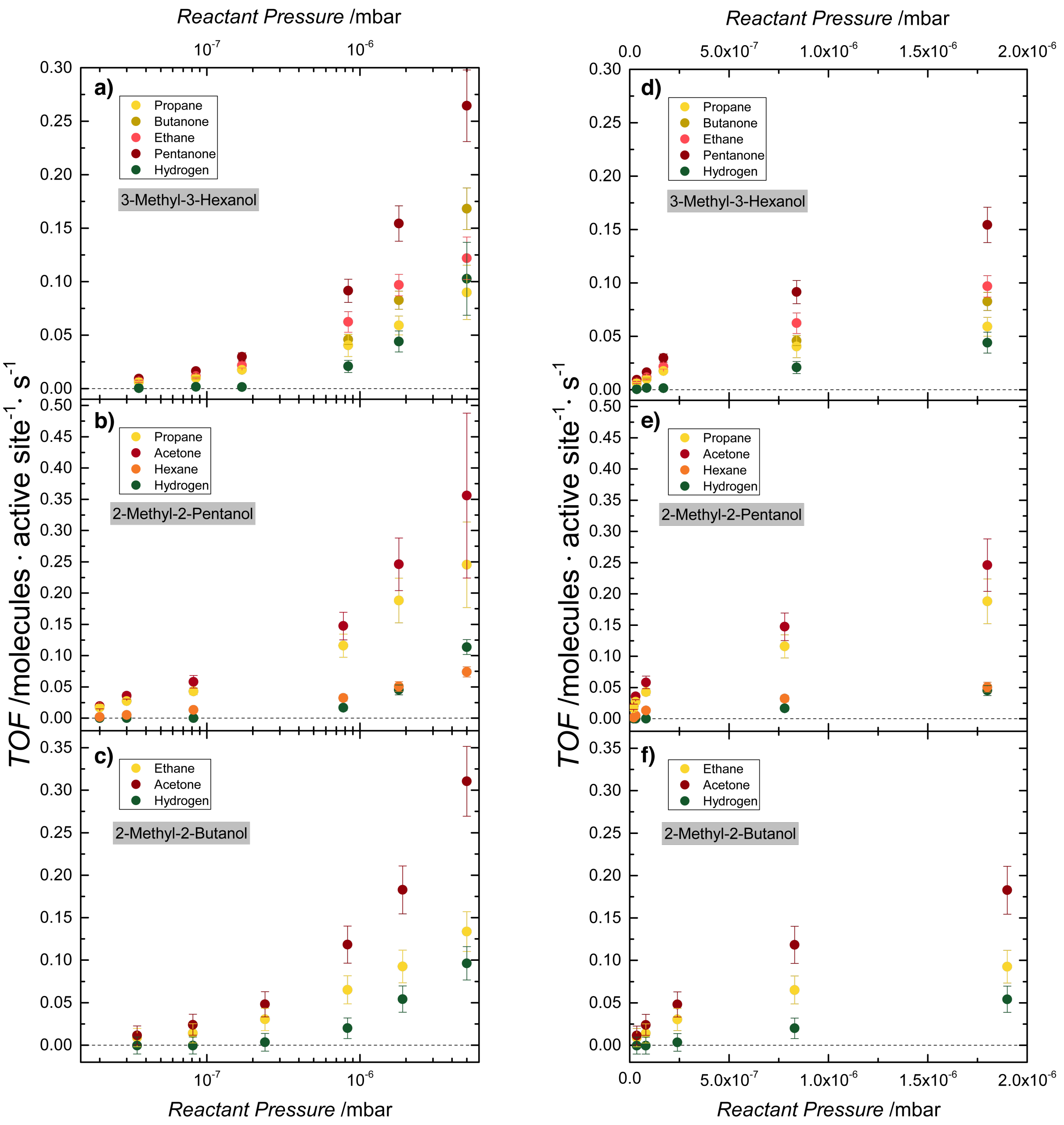

Figure S6: Pressure-dependent TOFs for photocatalytic reforming of **a)** and **d)** 3-methyl-3-hexanol, **b)** and **e)** 2-methyl-2-pentanol, **c)** and **f)** 2-methyl-2-butanol over 0.1% ML Pt_x/TiO_2. The catalytic experiments are performed at 336 K, so that the temperature is above the desorption temperature of the ketones and alkanes and below the desorption temperature of the alcohol. The TOFs exhibit a 1st order behaviour since the reaction is limited by reactant adsorption at 336 K. Due to higher temperature, a desorption limited regime is not reached, which is different to figure S5. In **a)**, **b)** and **c)**, the pressure-dependent TOFs are plotted on a logarithmic scale and in **d)**, **e)** and **f)**, a section of the TOFs are plotted linearly to demonstrate a linear rise with increasing alcohol pressure.

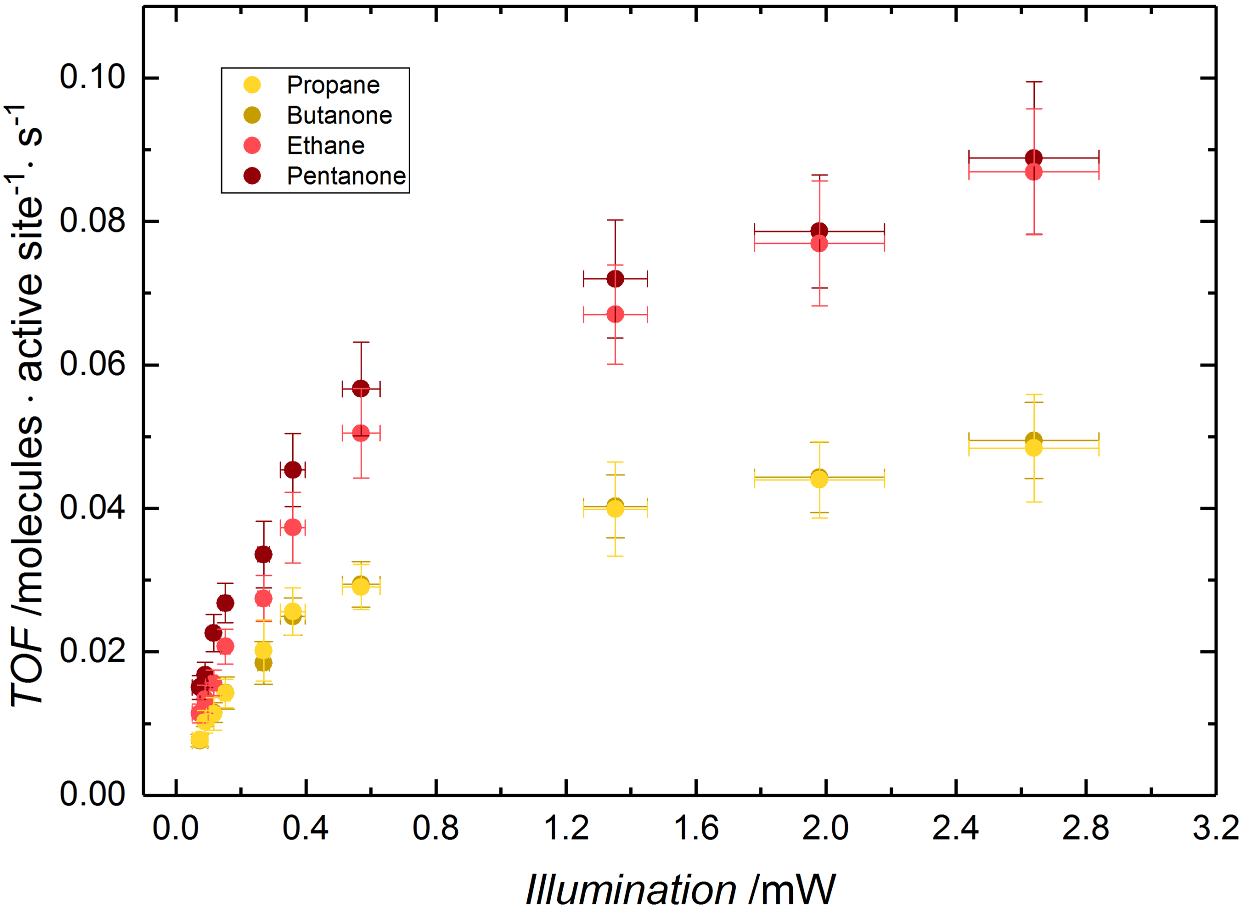

Figure S7: Power-dependent TOFs for photoreforming of 3-methyl-3-hexanol on 0.1% ML $Pt_x/TiO_2(110)$ at 336 K in an alcohol background pressure of $8.4 \cdot 10^{-7}$ mbar. The reaction exhibits a first-order dependence, which transfers into a zeroth-order regime at higher illumination intensities. This behavior, which is similar to the reaction of other alcohols,[S12] supplies evidence for a one photon process. Note that the overall apparent quantum yield ranges from 0.67 % for low illumination intensities (0.74 μW) to 0.11 % for higher photon fluxes (2.64 mW), when the calculations follow the generally applied procedure via the number of evolved molecules per second with respect to the photon flux[S12–S14] and assuming the usually assumed two-photon process for a direct comparison with literature values.

Selectivities

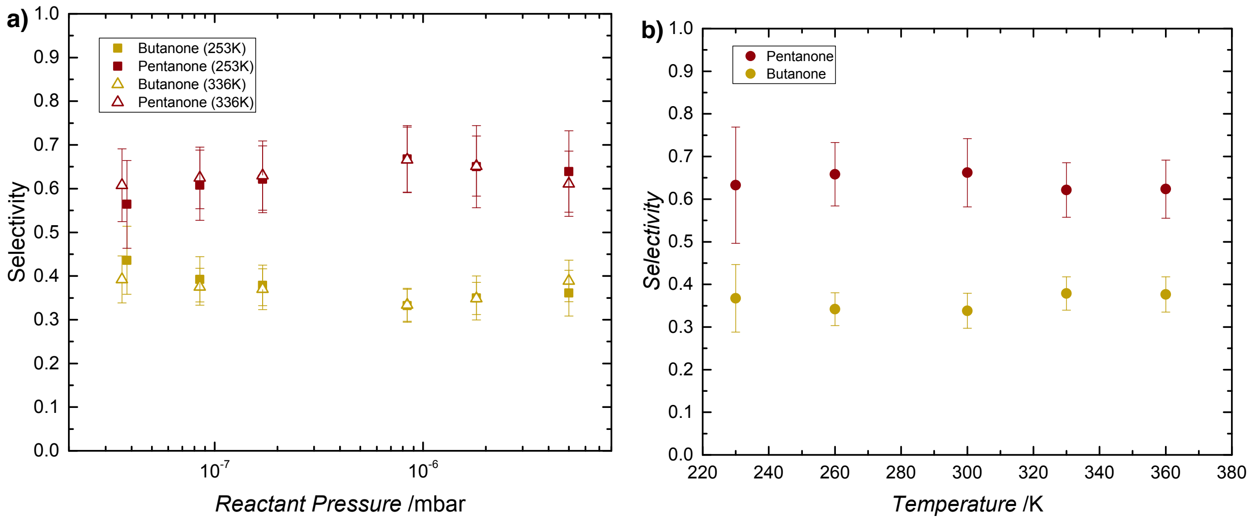

Figure S8: Photocatalytic conversion of 3-methyl-3-hexanol on 0.1% ML $Pt_x/TiO_2(110)$. In **a)**,the selectivities for 2-pentanone and 2-butanone based on the TOFs from figure S5 and figure S6 are displayed for different reactant pressures at two different temperatures 253 K and 336 K, respectively. The two temperatures typify the temperature regime limited by product desorption, respectively by reactant adsorption. In **b)**, the selectivities for 2-pentanone and 2-butanone are shown for different temperatures. It is found, that the selectivities are temperature and concentration independent.

QMS traces

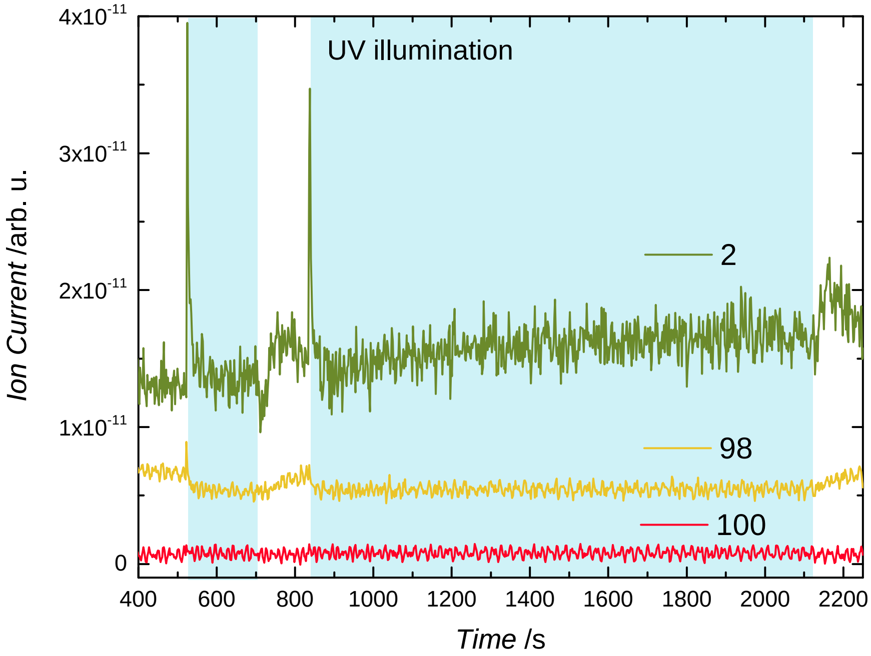

Figure S9: Photocatalytic alcohol reforming of 3-methyl-3-hexanol on $Pt_x/TiO_2(110)$ (0.1% ML cluster coverage). The masses $m/z = 2$ for hydrogen, $m/z = 98$ for the dehydration products, hexene and methylene hexane, and $m/z = 100$ for 3-hexanone are displayed at 340 K under a 3-methyl-3-hexanol pressure of $1.7 \cdot 10^{-7}$ mbar. The blue region highlights the period of UV irradiation. It can be clearly seen that neither of these products are quantitatively formed. Note that the decrease in the $m/z = 98$ during illumination is due to the consumption of the alcohol (the substrate), which exhibits a fragment of this particular mass in its fragmentation pattern.

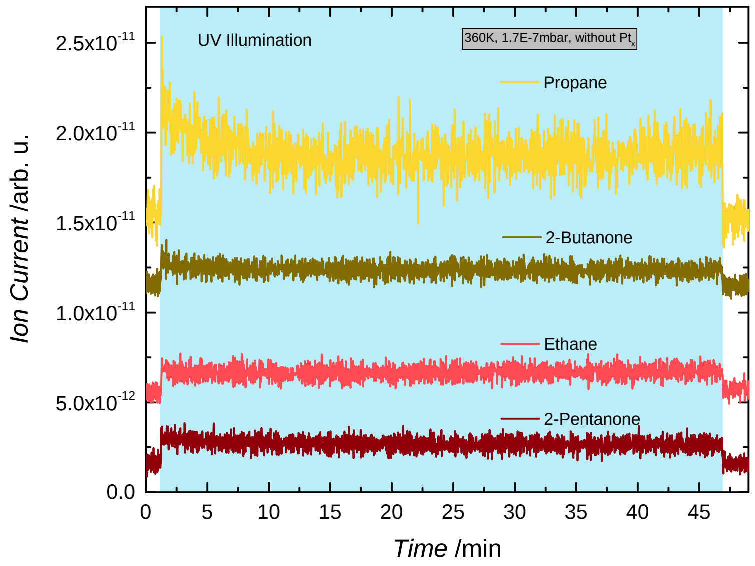

Figure S10: Photocatalytic products of 3-methyl-3-hexanol photoreforming on bare r−TiO_2(110). 3-methyl-3-hexanol (m/z=73), propane (m/z=29), 2-butanone (m/z=72), ethane (m/z=30), and 2-pentanone (m/z=86) signals are shown at 360 K under a 3-methyl-3-hexanol pressure of $1.7 \cdot 10^{-7}$ mbar. The blue region highlights the period of UV irradiation. Note that the traces are offset for clarity.

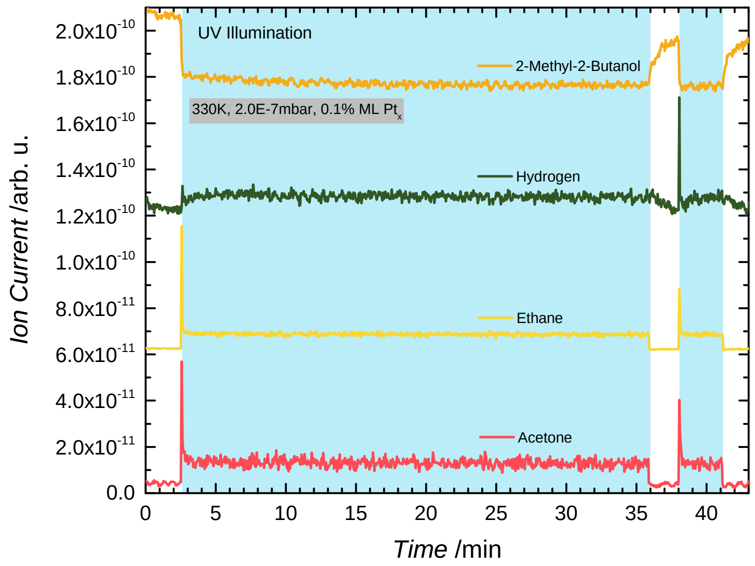

Figure S11: Photocatalytic products of 2-methyl-2-butanol photoreforming on 0.1% ML Pt_x/TiO_2(110). 2-methyl-2-butanol (m/z=73), hydrogen (m/z=2), ethane (m/z=30), and acetone (m/z=58) signals are shown at 330 K under a 2-methyl-2-butanol pressure of $2.0 \cdot 10^{-7}$ mbar. The blue region highlights the period of UV irradiation. Note that the traces are offset for clarity. Under these reactions conditions, a third reaction product next to acetone and ethane, namely hydrogen is observed.

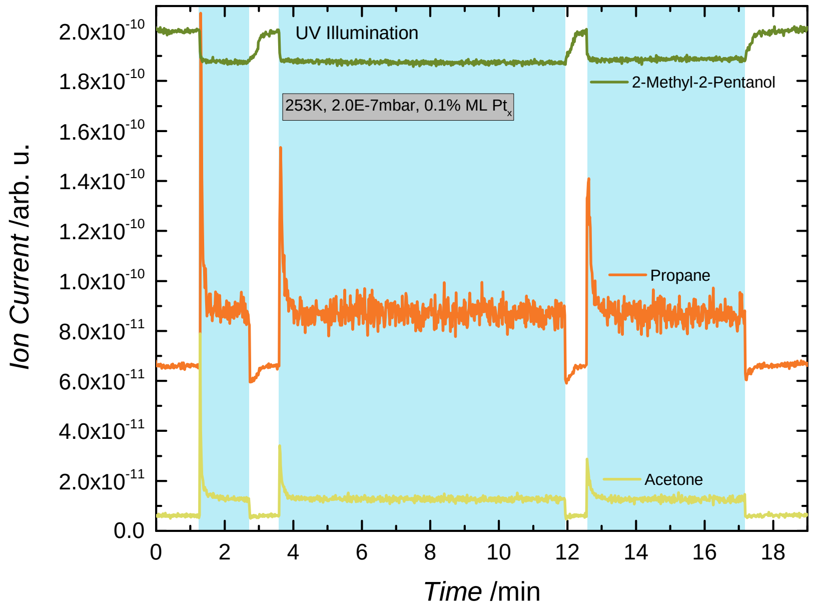

Figure S12: Photocatalytic products of 2-methyl-2-pentanol photoreforming on 0.1% ML $Pt_x/TiO_2(110)$. 2-methyl-2-pentanol (m/z=*87*), propane (m/z=*29*), and acetone (m/z=*58*) signals are shown at 253 K under a 2-methyl-2-pentanol pressure of $2.0 \cdot 10^{-7}$ mbar. The blue region highlights the period of UV irradiation. Note that the traces are offset for clarity.

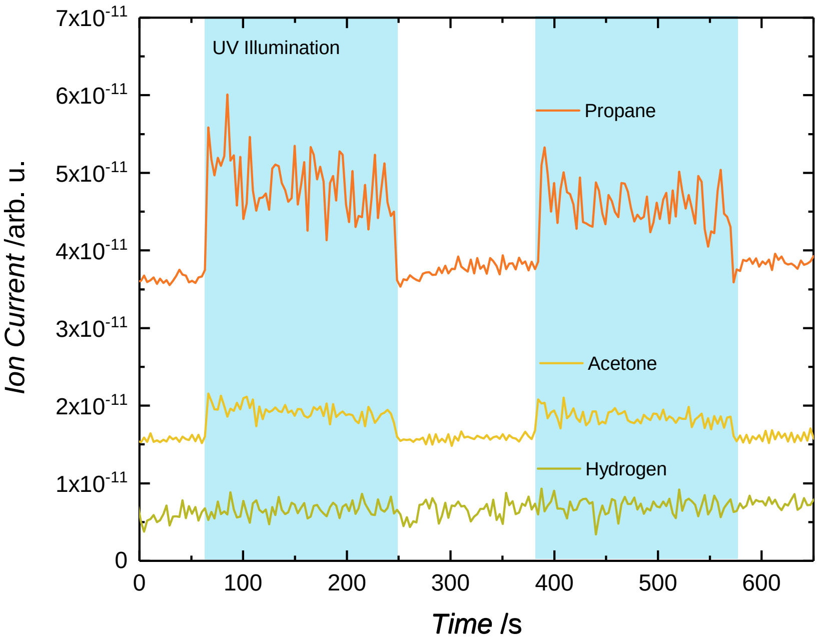

Figure S13: Photocatalytic products of 2-methyl-2-pentanol photoreforming on hydroxylated-TiO_2(110). Propane (m/z=29), acetone (m/z=58) and molecular hydrogen (m/z=2) signals are shown at 270 K under a 2-methyl-2-pentanol background pressure of $1.7 \cdot 10^{-7}$ mbar. The hydroxylated-TiO_2(110) crystal was exposed to water at cryogenic temperatures and annealed to 270 K. This leads to hydroxyl groups on the surface, while residual water molecules are being desorbed.[S15] The photoreaction occurs very similar to that on reduced-TiO_2(110) (see Fig. 3a) showing that the hydroxylation does not lead to significant changes in the reaction behavior. Note that the traces are offset for clarity.

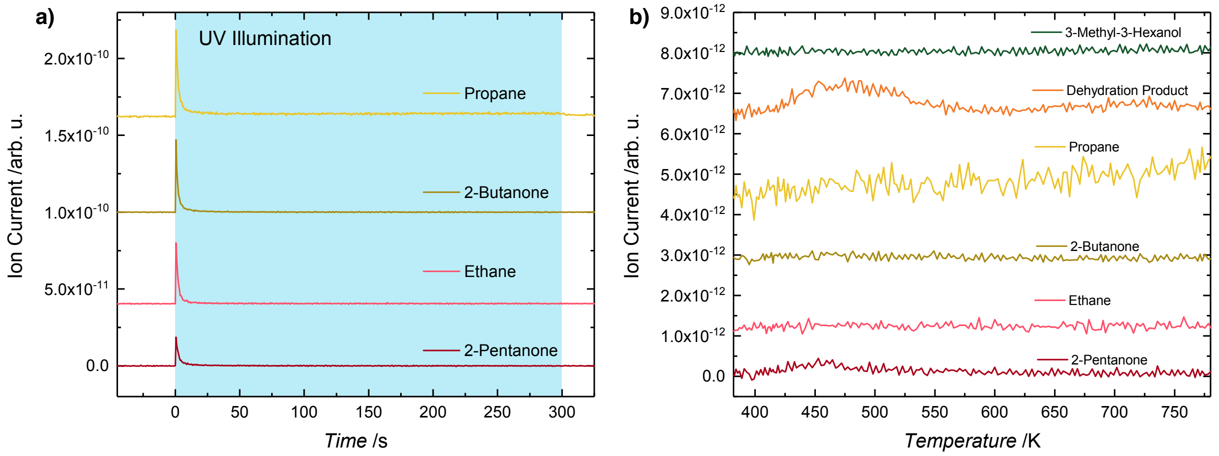

Figure S14: **a)** Isothermal photoreaction experiment of 5 L 3-methyl-3-hexanol on r-TiO_2(110) at 340 K. The catalyst is exposed to 3-methyl-3-hexanol at 150 K and then brought to 340 K. Upon illumination (blue region), propane (m/z=*44*), 2-butanone (m/z=*72*), ethane (m/z=*30*), and 2-pentanone (m/z=*86*) are formed.
b) Temperature programmed desorption spectroscopy (TPD) experiment after the isothermal photoreaction at 340 K. It is found that no alkanes and ketones appear in the TPD spectrum, which shows that the photoproducts have desorbed completely during illumination. Furthermore, no other photoproducts are detected. Only the formation of small amounts of dehydration products (m/z=*98*) are observed. They originate from the thermal water elimination of alcohol residues, a general property of the thermal reactivity of alcohols,[S16] and may lead to three different structural isomers for which an unambiguous assignment cannot be given by EI-QMS. (Note that in both plots the traces are offset for clarity.)

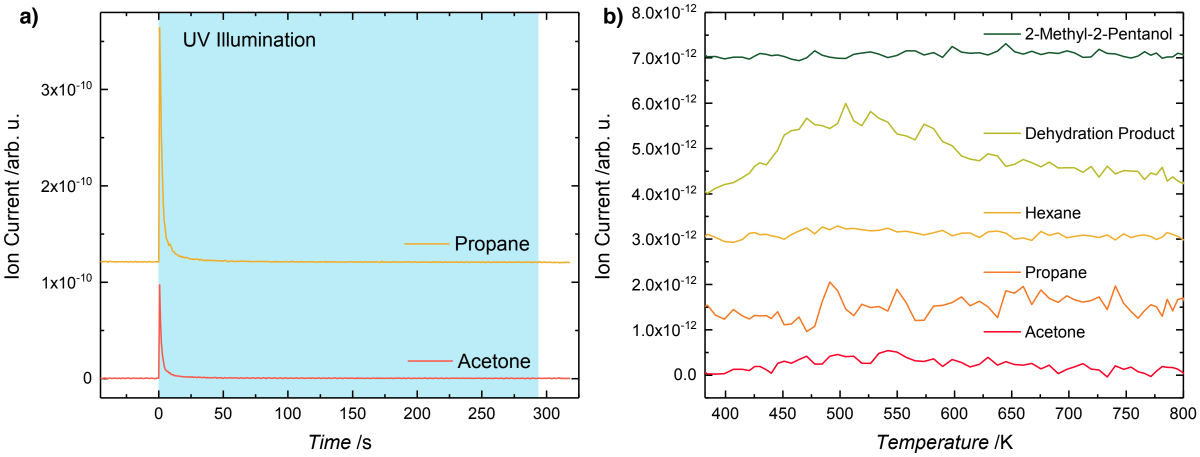

Figure S15: **a)** Isothermal photoreaction experiment of 5 L 2-methyl-2-pentanol on r-TiO_2(110) at 340 K. The catalyst is exposed to 2-methyl-2-pentanol at 150 K and then brought to 340 K. Upon illumination (blue region), propane (m/z=*29*) and acetone (m/z=*58*) are formed.

b) Temperature programmed desorption spectroscopy (TPD) experiment after the isothermal photoreaction at 340 K. It is found that acetone and propane do not appear in the TPD spectrum, which shows that the photoproducts have desorbed completely during illumination. Furthermore, no other photoproducts (e.g. product from alkyl recombination, i.e. hexane (m/z=*86*)) are detected. Only the formation of small amounts of dehydration products (m/z=*84*) are observed. They originate from the thermal water elimination of alcohol residues, a general property of the thermal reactivity of alcohols,[S16] and may lead to three different structural isomers for which an unambiguous assignment cannot be given by EI-QMS. (Note that in both plots the traces are offset for clarity.)

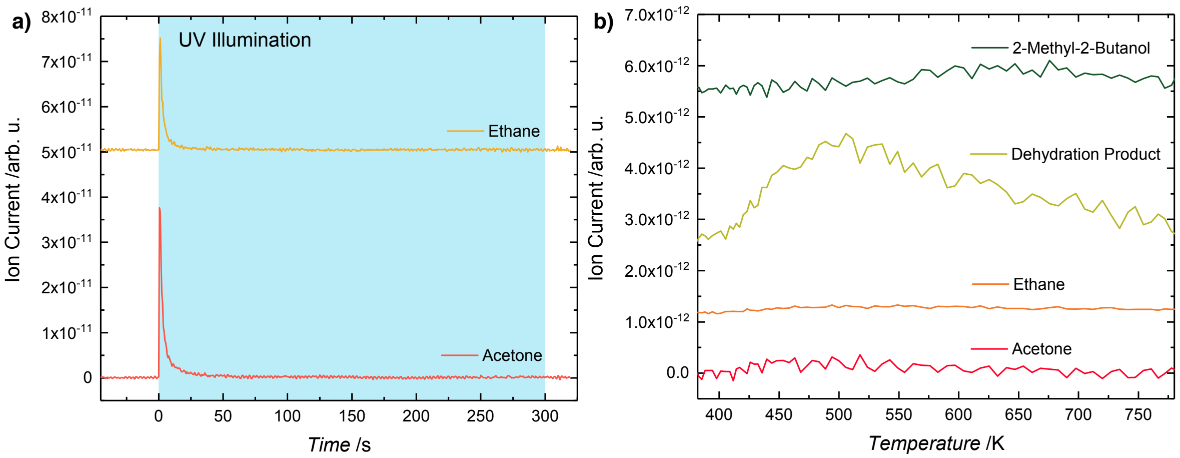

Figure S16: **a)** Isothermal photoreaction experiment of 5 L 2-methyl-2-butanol on r-TiO_2(110) at 340 K. The catalyst is exposed to 2-methyl-2-butanol at 150 K and then brought to 340 K. Upon illumination (blue region), ethane (m/z=*30*) and acetone (m/z=*58*) are formed.

b) Temperature programmed desorption spectroscopy (TPD) experiment after the isothermal photoreaction at 340 K. It is found that acetone and ethane do not appear in the TPD spectrum, which shows that the photoproducts have desorbed completely during illumination. Furthermore, no other photoproducts are detected. Only the formation of small amounts of dehydration products (m/z=*70*) are observed. They originate from the thermal water elimination of alcohol residues, a general property of the thermal reactivity of alcohols,[S16] and may lead to three different structural isomers for which an unambiguous assignment cannot be given by EI-QMS. (Note that in both plots the traces are offset for clarity.)

Auger Electron Spectroscopy

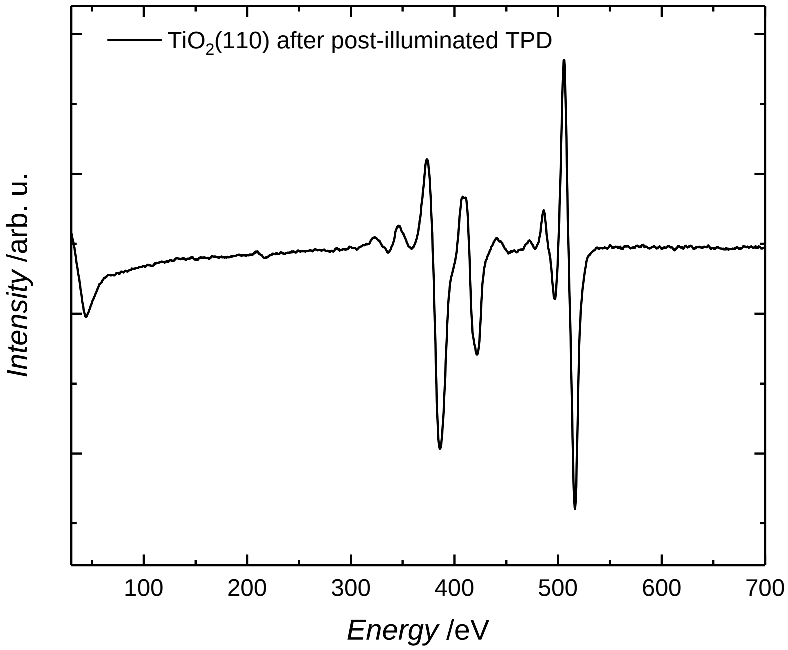

Figure S17: Auger Electron Spectrum of the reduced-TiO_2(110) after the isothermal photoreaction experiment of 5 L 3-methyl-3-hexanol at 340 K followed by a thermal programmed desorption from $340 - 800\,K$, shown in S14. No carbon containing species (expected at 272 eV) are detected.

Thermochemistry

Table S2: Standard enthalpy of formation (ΔH_f°) for molecules, which may be formed in different reactions.

Molecule	ΔH_f° [kJ/mol]
methane	-74.6[S17]
ethane	-84.0[S17]
propane	-103.8[S17]
butane	-125.6[S17]
pentane	-146.9[S17]
hexane	-167.1[S17]
acetone	-217.1[S17]
2-butanone	-238.5[S17]
2-pentanone	-259.0[S17]
3-hexanone	-277.6[S18]
2-methyl-2-butanol	-329.3[S18]
2-methyl-2-propanol	-352.1[S19]
3-methyl-3-hexanol	-372.8[S19]
atomic hydrogen	217.998[S17]
methyl radical	146.427[S20]
ethyl radical	119.87[S21]
propyl radical	100.87[S21]

Table S3: Standard enthalpy of reaction (ΔH_R°) calculated from the standard enthalpy of formation (ΔH_f°) of the respective reactants and products.

Reaction	ΔH_R° [kJ/mol]
2−methyl−2−butanol $\longrightarrow$ methane + 2−butanone	16.2
2−methyl−2−butanol $\longrightarrow$ ethane + acetone	28.2
2−methyl−2−butanol $\longrightarrow$ $\frac{1}{2}$ butane + acetone	98.8
2−methyl−2−propanol $\longrightarrow$ methane + 2−pentanone	18.5
2−methyl−2−propanol $\longrightarrow$ propane + acetone	31.2
2−methyl−2−propanol $\longrightarrow$ $\frac{1}{2}$ hexane + acetone	102.9
3−methyl−3−hexanol $\longrightarrow$ methane + 3−hexanone	20.6
3−methyl−3−hexanol $\longrightarrow$ ethane + 2−pentanone	29.8
3−methyl−3−hexanol $\longrightarrow$ propane + 2−butanone	30.5

All potential overall reactions are endothermic and elementary reaction steps are considered to require even more energy due to the cleavage of C-C bonds (see below).

The thermochemistry of three different model reactions is evaluated (see Table S4) in order to relate it to the selectivity of 3-methly-3-hexanol photoreforming. First, the required energy for a radical formation from the respective alkane via a C-H bond cleavage is calculated. Second, the scission of a C−C bond is addressed by the evaluation of the heat of reaction for the formation of two radicals from the respective alkane. Third, it is assumed that the reaction of the alcohol leads to a ketone, an alkyl radical and atomic hydrogen.

Table S4: Standard Enthalpy of Reaction (ΔH_R°) for three model reactions in order of evaluate the selectivity in 3-methyl-3-hexanol photoreforming (values calculated from Table S2).

Reaction	ΔH_R° [kJ/mol]
Model Reaction 1	
$CH_4 \longrightarrow CH_3^{\cdot} + H^{\cdot}$	439
$C_2H_6 \longrightarrow CH_3CH_2^{\cdot} + H^{\cdot}$	422
$C_3H_8 \longrightarrow CH_3CH_2CH_2^{\cdot} + H^{\cdot}$	423
Model Reaction 2	
$C_2H_6 \longrightarrow 2CH_3^{\cdot}$	377
$C_4H_{10} \longrightarrow 2CH_3CH_2^{\cdot}$	365
$C_6H_{14} \longrightarrow 2CH_3CH_2CH_2^{\cdot}$	369
Model Reaction 3	
$3-methyl-3-hexanol \longrightarrow 3-hexanone + CH_3^{\cdot} + H^{\cdot}$	460
$3-methyl-3-hexanol \longrightarrow 2-pentanone + CH_3CH_2^{\cdot} + H^{\cdot}$	452
$3-methyl-3-hexanol \longrightarrow 2-butanone + CH_3CH_2CH_2^{\cdot} + H^{\cdot}$	453

The reactions yielding a methyl radical require significantly more energy than those for the other two radicals. Reactions for the formation of propyl and ethyl radicals are very similar in energy, but in every case the formation of ethyl is energetically favoured over that of propyl. This is the same trend as for the observed selectivity in 3-methly-3-hexanol photoreforming.

References

(S1) Kollmannsberger, S. L.; Walenta, C. A.; Courtois, C.; Tschurl, M.; Heiz, U. Thermal Control of Selectivity in Photocatalytic, Water-Free Alcohol Photoreforming. *ACS Catal.* **2018**, *8*, 11076 – 11084.

(S2) Heiz, U.; Vanolli, F.; Trento, L.; Schneider, W.-D. Chemical Reactivity of Size-Selected Supported Clusters: An Experimental Setup. *Review of Scientific Instruments* **1997**, *68*, 1986–1994.

(S3) Walenta, C. A.; Kollmannsberger, S. L.; Kiermaier, J.; Winbauer, A.; Tschurl, M.; Heiz, U. Ethanol Photocatalysis on Rutile TiO_2(110): the Role of Defects and Water. *Phys. Chem. Chem. Phys.* **2015**, *17*, 22809–22814.

(S4) Henderson, M. A. Structural Sensitivity in the Dissociation of Water on TiO_2 Single-Crystal Surfaces. *Langmuir* **1996**, *12*, 5093–5098.

(S5) Geng, Z.; Jin, X.; Wang, R.; Chen, X.; Guo, Q.; Ma, Z.; Dai, D.; Fan, H.; Yang, X. Low-Temperature Hydrogen Production via Water Conversion on Pt/TiO_2. *J. Phys. Chem. C* **2018**, *122*, 10956–10962.

(S6) Isomura, N.; Wu, X.; Hirata, H.; Watanabe, Y. Cluster Size Dependence of Pt Core-Level Shifts for Mass-Selected Pt Clusters on TiO_2(110) Surfaces. *J. Vac. Sci. Technol., A* **2010**, *28*, 1141–1144.

(S7) Watanabe, Y.; Isomura, N. A New Experimental Setup for High-Pressure Catalytic Activity Measurements on Surface Deposited Mass-Selected Pt Clusters. *J. Vac. Sci. Technol., A* **2009**, *27*, 1153–1158.

(S8) Isomura, N.; Wu, X.; Watanabe, Y. Atomic-Resolution Imaging of Size-Selected Platinum Clusters on TiO_2(110) Surfaces. *J. Chem. Phys.* **2009**, *131*.

(S9) Bonanni, S.; Aït-Mansour, K.; Harbich, W.; Brune, H. Effect of the TiO_2 Reduction State on the Catalytic CO Oxidation on Deposited Size-Selected Pt Clusters. *J. Am. Chem. Soc.* **2012**, *134*, 3445–3450.

(S10) Harrison, A. G.; Jones, E. G.; Gupta, S. K.; Nagy, G. P. Total Cross Sections for Ionization by Electron Impact. *Can. J. Chem.* **1966**, *44*, 1967–1973.

(S11) Kim, Y.-K. et al. Electron-impact ionization cross section for ionization and excitation database. `http://physics.nist.gov/ionxsec`, 2004 version 3.0; 2018, February 12.

(S12) Walenta, C. A.; Kollmannsberger, S. L.; Courtois, C.; Pereira, R. N.; Stutzmann, M.; Tschurl, M.; Heiz, U. Why Co-Catalyst-Loaded Rutile Facilitates Photocatalytic Hydrogen Evolution. *Phys. Chem. Chem. Phys.* **2019**, *21*, 1491–1496.

(S13) Kudo, A.; Miseki, Y. Heterogeneous Photocatalyst Materials for Water Splitting. *Chem. Soc. Rev.* **2009**, *38*, 253 – 278.

(S14) Hisatomi, T.; Kubota, J.; Domen, K. Recent Advances in Semiconductors for Photocatalytic and Photoelectrochemical Water Splitting. *Chem. Soc. Rev.* **2014**,

(S15) Kim, B.; Li, Z.; Kay, B. D.; Dohnalek, Z.; Kim, Y. K. The Effect of Oxygen Vacancies on the Binding Interactions of NH_3 with Rutile TiO_2(110)-(1x1). *Phys. Chem. Chem. Phys.* **2012**, *14*, 15060–15065.

(S16) Kim, Y. K.; Kay, B. D.; White, J. M.; Dohnalek, Z. Alcohol Chemistry on Rutile TiO_2(110) The Influence of Alkyl Substituents on Reactivity and Selectivity. *J. Phys. Chem. C* **2007**, *111*, 18236–18242.

(S17) Dean, J. A.; Lange, N. A. *Lange's Handbook of Chemistry, 15th Edition*; New York: McGraw-Hill, 1999.

(S18) CRC Handbook, In *CRC Handbook of Chemistry and Physics, 85th Edition*; Lide, David R.,, Ed.; CRC Press, 2004.

(S19) Alberty, R. A.; Chung, M. B.; Flood, T. M. Standard Chemical Thermodynamic Properties of Alkanol Isomer Groups. *J. Phys. Chem. Ref. Data* **1987**, *16*, 391.

(S20) Baghal-Vayjooee, M. H.; Colussi, A. J.; Benson, S. W. Very Low Pressure Reactor. A New Technique for Measuring Rates and Equilibria of Radical-Molecule Reactions at Low Temperature. Heat of Formation of the Methyl Radical. *J. Am. Chem. Soc.* **1978**, *100*, 3214–3215.

(S21) Houle, F. A.; Beauchamp, J. L. Photoelectron Spectroscopy of Methyl, Ethyl, Isopropyl, and *tert*-Butyl Radicals. Implications for the Thermochemistry and Structures of the Radicals and Their Corresponding Carbonium Ions. *J. Am. Chem. Soc.* **1979**, *101*, 4067–4074.
